# Valorization of *Moringa oleifera* Lam.: Healthy green biomass for circular bioeconomy

**DOI:** 10.1016/j.fochx.2025.102358

**Published:** 2025-03-09

**Authors:** Harsh Kumar, Shivani Guleria, Rajni Dhalaria, Eugenie Nepovimova, Nidhi Bhardwaj, Pooja Jha, Daljeet Singh Dhanjal, Narinder Verma, Tabarak Malik

**Affiliations:** a Centre of Advanced Technologies, Faculty of Science*,* University of Hradec Kralove*,* Rokitanskeho 62, 50003 Hradec Kralove*,* Czech Republic; b Department of Biotechnology, TIFAC-Centre of Relevance and Excellence in Agro and Industrial Biotechnology (CORE)*,* Thapar Institute of Engineering and Technology*,* Patiala 147001*,* India; c School of Biological and Environmental Sciences*,* Shoolini University of Biotechnology and Management Sciences*,* Solan 173229*,* India; d Department of Chemistry, Faculty of Science*,* University of Hradec Kralove*,* 50003 Hradec Kralove*,* Czech Republic; e Centre for Advanced Innovation Technologies, VSB-Technical University of Ostrava, 70800 Ostrava-Poruba*,* Czech Republic; f Department of Food Engineering and Technology*,* Institute of Chemical Technology*,* Mumbai 400019*,* India; g School of Bioengineering and Biosciences*,* Lovely Professional University*,* Phagwara*,* Punjab 144411*,* India; h School of Business Management, Shoolini University of Biotechnology and Management Sciences, Solan 173229, India; i Department of Biomedical Sciences, Institute of Health*,* Jimma University*,* Ethiopia; j Division of Research and Development, Lovely Professional University, Phagwara, Punjab 144401, India

**Keywords:** *Moringa oleifera*, Underutilized crop, Polyphenols, Polysaccharides, Functional biomaterials, Safety

## Abstract

Exploration of plant biodiversity that not only withstand extreme environmental conditions but also has the potential to fulfil sustainable development goals (SDGs) is the priority for researchers. *Moringa oleifera* is the best-suited plant in this category. It plays a primary role in SDGs due to its versatile features like health-beneficial effects. The polyphenols found in the different parts of this plant have exhibited health-promoting benefits and served as catalysts/resources for producing valuable ingredients. The current review outlines the potential application of *Moringa oleifera* in biofuel production, the synthesis of green nanomaterials, and the fortification of functional foods and feed to enhance nutritional value. Besides that, the application of *Moringa oleifera* in pharmaceutical products and the safety considerations associated with its utilization have also been examined. Conclusively, the review comprehensively aligns towards sustainable practices in the agro-industrial sector alongside the circular bioeconomy concept.

## Highlights

• *Moringa oleifera* has come under the category of neglected and underutilized species.
• *M. oleifera* is a rich source of phytochemicals associated with health activities.
• Valorization of *M. oleifera* different parts has been producing remarkable functional products.
• Systematic employment of *M. oleifera* plays an integral part in the circular bioeconomy.

## 1 Introduction

**Fig. 1:**
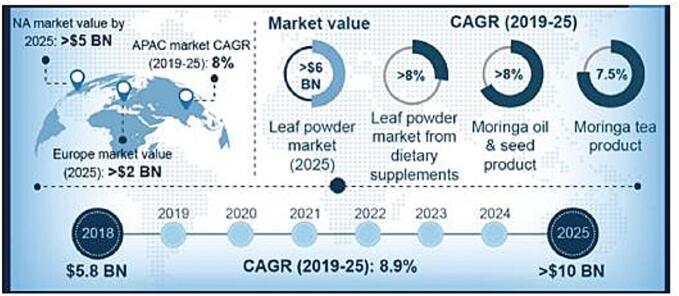
Market status of *Moringa* (Islam et al., 2021, Creative Commons Attribution License).

Neglected and underutilized crops provide enormous benefits across multiple domains, from increasing rural livelihoods to human health and nutrition, with potential support for agricultural biodiversity conservation (Bilali et al., 2024). Mabhaudhi et al. (2016) further contended that the promotion of these neglected and underutilized species (NUS) might take center stage to achieve sustainable development goals (SDGs). The SDGs include hunger elimination, reducing poverty, enhancing human health and well-being, and preserving terrestrial ecosystems. Bilali et al. (2024) argued that for the full realization of NUS in developing countries, further research on the utilization of NUS is required. In this context, their study reported *Moringa oleifera* as a key example of a critical NUS product. *Moringa oleifera* is a fast-growing deciduous leaf-shedding species. The species originates from the Indian subcontinent and belongs to the family *Moringa*ceae (Bilali et al., 2024). Although it is not very well-known in industrialized countries, *Moringa* has enormous possibilities for various industrial applications (Trigo et al., 2021). According to Němec et al. (2020), 705 *Moringa* trees have a plantation that provides sufficient leaf biomass to meet the nutritional needs of 340 adults for one meal regardless of gender. The nutrient profile makes *M. oleifera* stand out since it is instrumental in dealing with hunger and poverty. It creates employment among people from poor backgrounds, which helps boost the economy (Trigo et al., 2020). *Moringa* has been widely referred to as the “Miracle tree” and “Mother's best friend” due to its medicinal properties, which can help in treating and curing more than 300 diseases. The tree is also known to be very resistant, surviving in dry conditions. The leaves, seeds, roots, bark, fruits, seedpods, gum, oil, and flowers of *M. oleifera* provide great potential environmental applications and health benefits for humans and animals alike (Pop et al., 2022). Dubbed as a “superfood,” *Moringa* is famous for its exemplary nutritional profile, as shown in Table 1. It is rich in phytochemicals and other bioactive organic compounds, contributing to the diverse range of properties that make it healthy. These bioactive compounds are the most suitable for formulations in pharmaceutical products with minimal side effects and synergistic health benefits. The market for *Moringa* supplements is expected to exceed USD 6 billion by 2025 based on increasing demand for its incorporation in food products (Islam et al., 2021). Fig. 1 provides an overview of Moringa's current market landscape. *Moringa* cultivation is considered a triple-bottom-line approach, which focuses on sustainable production methods that enhance the health of soil via natural practices (Horn et al., 2022).

**Table 1:** Nutritional profile of different parts of *Moringa oleifera* Lam.

Nutrients	Fresh leaf	Nutrients	Seed	Nutrients	Flower	Nutrients	Stem	Nutrients	Root	Nutrients	Pod
Energy (Kcal/g)	286.9	Energy (Kcal/g)	564.5	Energy (Kcal/g)	391.2	Energy (Kcal/g)	380.05	Energy (Kcal/g)	384.05	Energy (Kcal/g)	178.2
Moisture (g/100 g)	74.3–76.3	Moisture (g/100 g)	4.7–5	Moisture (%)	10.4	Moisture (%)	5.5	Moisture (%)	6.9	Moisture (g/100 g)	80.1–85.7
Crude protein (g/100 g)	18–20.9	Crude protein (g/100 g)	26–26.9	Crude protein (g/100 g)	18.9	Crude protein (%)	3.59	Crude protein (%)	5.02	Crude protein (g/100 g)	81.1–85.7
Ash (g/100 g)	3.1–3.6	Ash (g/100 g)	5.2–5.8	Ash (g/100 g)	9.7	Ash (%)	1.6	Ash (%)	4.9	Ash (g/100 g)	12.3–13.2
Carbohydrates (g/100 g)	37.8	Carbohydrates (g/100 g)	14.4	Carbohydrates (g/100 g)	36	Carbohydrates (%)	87.4	Carbohydrates (%)	76.7	Carbohydrates (g/100 g)	26.4
Crude fibers (g/100 g)	7.7	Crude fibers (g/100 g)	4.9	Crude fibers (g/100 g)	–	Crude fibers (g/100 g)	0.4	Crude fibers (g/100 g)	0.8	Crude fibers (g/100 g)	35
Minerals (mg/100 g) Calcium Phosphorus Magnesium Potassium Sodium Zinc Manganese Copper Iron	738.9 89.8 147.6 494.1 21.9 1.2 13.5 1.2 16	Minerals (mg/100 g) Calcium Phosphorus Magnesium Potassium Sodium Zinc Manganese Copper Iron	76.8 524.3 259.8 64.2 24.9 27.5 87.7 48.1 13.7	Minerals (mg/100 g) Calcium Phosphorus Magnesium Potassium Sodium Zinc Manganese Copper Iron	2.3 - - 3 120.9 - - - -	Minerals (mg/100 g) Calcium Phosphorus Magnesium Potassium Sodium Zinc Manganese Copper Iron	1.3 - - 32.4 378.3 - - - -	Minerals (mg/100 g) Calcium Phosphorus Magnesium Potassium Sodium Zinc Manganese Copper Iron	3.9 - - 15.4 514.8 - - - -	Minerals (mg/100 g) Calcium Phosphorus Magnesium Potassium Sodium Zinc Manganese Copper Iron	29 112.3 25.2 263.5 15.3 0.3 8.4 3.2 5.3
Amino acids (g/100 g) Lysine Histidine Arginine Threonine Valine Methionine Isoleucine Leucine Phenylalanine Aspartate Serine Glutamate Proline Glycine Alanine Cysteine Tyrosine	4.4 2.2 4.8 2.2 4.8 1.2 4.2 7 4 6.8 3 9 3 3.4 4.1 0.6 3.1	Amino acids (g/100 g) Lysine Histidine Arginine Threonine Valine Methionine Isoleucine Leucine Phenylalanine Aspartate Serine Glutamate Proline Glycine Alanine Cysteine Tyrosine	4.2 2.1 5 3 3 1 2.3 6.8 3.5 7 2.6 10.5 2.5 3 4.9 0.5 3	Amino acids (g/100 g) Lysine Histidine Arginine Threonine Valine Methionine Isoleucine Leucine Phenylalanine Aspartate Serine Glutamate Proline Glycine Alanine Cysteine Tyrosine	3 1.1 6.5 2 2 0.5 2.1 5.2 3.9 4 2.8 7.2 2.3 1 4 0.6 1.5	Amino acids (g/100 g) Lysine Histidine Arginine Threonine Valine Methionine Isoleucine Leucine Phenylalanine Aspartate Serine Glutamate Proline Glycine Alanine Cysteine Tyrosine	2.6 1 2.1 1 4.3 0.7 2 4.5 3.7 3.1 2.4 5 3.1 2.4 3.8 0.5 2.5	Amino acids (g/100 g) Lysine Histidine Arginine Threonine Valine Methionine Isoleucine Leucine Phenylalanine Aspartate Serine Glutamate Proline Glycine Alanine Cysteine Tyrosine	2.9 0.5 3.6 2.1 1 1 0.8 1 1 0.9 0.9 8 0.5 0.7 5 1.2 1	Amino acids (mg/g) Lysine Histidine Arginine Threonine Valine Methionine Isoleucine Leucine Phenylalanine Aspartate Serine Glutamate Proline Glycine Alanine Cysteine Tyrosine	2.5 2 8.1 3.3 4.3 0.9 3.1 5.6 2.3 7.4 7.5 14.6 4 4.3 4.2 - 0.4
Vitamins (mg/100 g) Thiamine (B1) Riboflavin (B2) Niacin (B3) Ascorbic acid (C) Tocopherol (E)	0.06 0.05 0.8 220 448	Vitamins (mg/100 g) Thiamine (B1) Riboflavin (B2) Niacin (B3) Ascorbic acid (C) Tocopherol (E)	0.05 0.06 0.2 4.5 751.6	Vitamins (mg/100 g) Thiamine (B1) Riboflavin (B2) Niacin (B3) Pyridoxine (B6) Ascorbic acid (C) Tocopherol (E)	- - - 7.69 459.1 -	Vitamins (mg/100 g) Thiamine (B1) Riboflavin (B2) Niacin (B3) Ascorbic acid (C) Tocopherol (E)	- - 1.3 71.4 -	Vitamins (mg/100 g) Thiamine (B1) Riboflavin (B2) Niacin (B3) Ascorbic acid (C) Tocopherol (E)	- - 5.8 48.1 -	Vitamins (mg/100 g) Thiamine (B1) Riboflavin (B2) Niacin (B3) Ascorbic acid (C) Tocopherol (E)	0.05 0.07 0.2 120 -

Source: Chhikara et al., 2021; Igwilo et al., 2017; Gopalakrishnan et al., 2016; Olaofe et al., 2013; Sánchez-Machado et al., 2010

Most conservation projects aim to restore native forests, storing carbon while hosting some biodiversity (Horn et al., 2022). Further, the cultivation of *Moringa* in rural communities is deemed to generate employment possibilities and income-generating activities by utilising the plant's parts for versatile applications (Horn et al., 2022). The use of *Moringa* biomass reduces greenhouse gas emissions into the atmosphere. With its nutritional sensitivity and climate-resilient characteristics, *Moringa* fits well in establishing a sustainable bio-economy (Horn et al., 2022). The massive cultivation of *Moringa* mitigates the negative impact of climate change and variability while addressing food and nutrition insecurity in order to improve the quality of life, especially for the most vulnerable members of society (Horn et al., 2022). *Moringa's* resilience and nutritional advantages could solve interconnected global challenges if its full potential is harnessed.

Circular bioeconomy integrates concepts like reduction, reuse, recycling, and recovery of materials. At the same time, nexus thinking highlights the interconnected use of waste via the synergies among energy resources, food and water (Parsa et al., 2021). These frameworks have gained considerable attention for their potential to facilitate the achievement of SDGs (Albrecht et al., 2018). The central principles of the circular economy have increasingly drawn the interest of governments, policymakers, scholars, and public communities as a feasible alternative to the conventional linear economy model. The lack of resources is a significant factor prompting the adaptation of the circular bioeconomy practices. When implemented effectively can result in significant decrease in raw materials consumption and waste generation, thereby advancing SDGs associated with environment sustainability, human health, and water and sanitation (Giannetti et al., 2020). In recent years, industries have witnessed a global transition aimed at achieving zero-emission goals, which are essential for achieving circularity (Orejuela-Escobar et al., 2021). The agricultural and industrial sectors were recognized as significant contributor at the global level for biowaste emissions, and the reducing of agro-industrial biowaste has become the priority for effective implementation of the circular practices (Mohan & Katakojwala, 2021). The review emphasizes the potential of *M. oleifera* in achieving multiple SDGs, viz., affordable clean energy, clean water and sanitation, decent work and economic growth, good health and well-being, no poverty and zero hunger. This study aims to investigate the use of *M. oleifera* green biomass in varied applications; therefore, with this concept, we have established diverse objectives to encompass the significant applied areas. The first objective of this study is to explore the advantages of green metallic nanoparticles derived from various *M. oleifera* biomass sources, emphasizing their broad applications across industries and therapeutic areas. The study's second objective highlights the use of biochar and bio-adsorbents derived from *M. oleifera,* underlining their impactful roles in agriculture and environmental management. Third, this study investigates the potential of *M. oleifera-*derived biodiesel as a sustainable, eco-friendly energy source. The fourth objective examines the effective incorporation of *M. oleifera* biomasses to enhance the quality of various food products, such as cereal-based foods, other food items, and edible films or coatings. Fifth, the study evaluates the application of *M. oleifera*-based additives in animal feed, specifically for chickens, fish, pigs, and grazing livestock. The sixth objective looks at herb treatments processed from *M. oleifera* biomass. Finally, the review touches on major safety concerns associated with the use of *M. oleifera*, offering an integrated discussion of its advantages and potential uses. All above mentioned SDGs have been covered up in the coming sections and sub-sections.

## 2 General overview of the polyphenols and polysaccharides in *M. oleifera*

Polyphenols constitute a class of secondary metabolites ubiquitous and widespread throughout the plant kingdom (Yang et al., 2023). The polyphenolic compounds in *M. oleifera* leaves include flavonoids and phenolic acids (Jabar et al., 2022; Yang et al., 2023). The total phenolic content in the leaves has been measured to vary between 32.93 and 1417 mg of gallic acid equivalents (GAE) per 100 g of fresh weight (FW). Furthermore, HPLC analysis revealed that hydroxybenzoic acid derivatives were the dominant phenolic compounds in leaves at 73.475 mg/100 g DW. This would mean approximately 21 % of phenolic content (Teclegeorgisha et al., 2021). Hydroxycinnamic acid derivatives accounted for 63.612 mg/100 g DW, thus relating to 18 % of the total phenolic compounds. Other significant contributors include rutin (85.15 mg/100 g DW), quercetin pentosidine (80.613 mg/100 g DW), chlorogenic acid (52.629 mg/100 g DW), quercetin derivatives (49.87 mg/100 g DW), and shamrock phenol derivatives (16.11 mg/100 g DW). The two compounds combined make up approximately 55 % of the total phenolic content within the leaves (Yang et al., 2023). Research has also indicated that different solvents, including deionized water, ethyl acetate, n-butanol, and n-hexane, can be used to extract flavonoids from *Moringa oleifera* leaves. Specifically, extraction with ethyl acetate produced a flavonoid composition of over 89 %. On the other hand, the total flavonoid content extracted with 70 % ethanol was found to contain 6.2 g/100 g (Manaheji et al., 2011). The major polyphenols present in *M. oleifera* leaves are shown in Table 2. Prabakaran et al. (2018) analyzed the different types of phenolic compounds in Moringa using UHPLC. Results established that the significant phenolic compounds from different parts of the *Moringa* plant, such as bark, flowers, leaves, roots, and seeds, were flavonols quercetin, myricetin, and hydroxybenzoic acids. The percentage of myricetin in the leaves of *Moringa oleifera* stands as high as 1530 ± 10 μg/g, while quercetin stays at the second position at 985 ± 4 μg/g. Meanwhile, the roots contained considerable amounts of biochanin A at 45 ± 1 μg/g and gentisic acid at 85 ± 2 μg/g. Ravani et al. (2018) mainly focused their attention on the phenolic content present in the pulp of *Moringa* pods. They isolated high levels of total phenolics in *Moringa* pod pulp. Total phenols were found to be 11.02 mg of GAE/g, with a high amount of flavonoids, of which quercetin was found at the level of 3.42 mg of QAE/g. Singh et al. (2013) determined free phenolic acid and flavonoid content in defatted *M. oleifera* seed flour by the HPLC method. They reported that bound phenolic compound extractability was much higher at *p* < 0.05 compared to free phenolic extracts, which had an amount of 4173.00 ± 32.22 mg GAE/100 g, whereas that of the freeextracts were 780.00 ± 14.2 mg GAE/100 g. Ten phenolic compounds, including caffeic acid, catechin, cinnamic acid, epicatechin, ferulic acid, gallic acid, p-coumaric acid, protocatechuic acid, quercetin, and vanillin were successfully isolated and quantified by the study (Table 2).

**Table 2:** Polyphenols in the different parts of *Moringa oleifera* Lam.

Leaf	Seed	Flower	Stem	Root	Pod	References
**Flavonoids:** Quercetin, isoquercetin, kaempferol, naringenin, naringin, catechin, myricetin	**Flavonoids:** Quercetin, isoquercetin, kaempferol, naringenin, naringin, catechin, myricetin	**Flavonoids:** Quercetin, kaempferol, naringenin, naringin, catechin, myricetin	**Flavonoids:** Quercetin, isoquercetin, kaempferol, naringenin, naringin, catechin, myricetin, procyanidins	**Flavonoids:** Quercetin, kaempferol, naringenin, naringin, catechin, myricetin, procyanidins	**Flavonoids:** Caohuoside D, ambofuracin	Prabakaran et al., 2018; Rani et al., 2018; Ravani et al., 2018; Amin et al., 2024
**Phenolic acids:** O coumaric acid, gallic acid, ellagic acid, ferulic acid, caffeic acid, sinapic acid, chlorogenic acid	**Phenolic acids:** O-coumaric acid, gallic acid, ferulic acid, caffeic acid, cinnamic acid	**Phenolic acids:** O-coumaric acid	**Phenolic acids:** O-coumaric acid	**Phenolic acids:** O-coumaric acid	**Phenolic acids:** -	Singh et al., 2013; Prabakaran et al., 2018; Rani et al., 2018
**Stilbenoids:** Resveratrol	**Stilbenoids:** Resveratrol	**Stilbenoids:** Resveratrol	**Stilbenoids:** Resveratrol	**Stilbenoids:** Resveratrol	**Stilbenoids:** -	Prabakaran et al., 2018

Polysaccharides are highly functional and are among the most nutritious and therapeutically valuable compounds in *M. oleifera* (Yang et al., 2022). That is why there has been growing interest in extracting polysaccharides from different parts of the *M. oleifera*. Polysaccharides from *Moringa oleifera* (MOPs) have been reported to include various biological activities such as antibacterial, antidiabetic, antitumor, and immune-modulating effects (Singhal et al., 2012). MOPs are polar macromolecules, mainly insoluble in organic solvents and slightly soluble in cold water (Table 3). Pramanik and Islam (1998) were the first to extract and purify polysaccharides from *Moringa* pods and blossoms, which provided a basis for the subsequent studies on *Moringa* polysaccharides. In their pioneering study, they focused on the extraction and characterization of polysaccharides from immature flowers. It was found that d-glucose, D-galactose, and D-glucuronic acid are the predominant monosaccharides presented in the molar ratio of 1:1.9:0.9. These monosaccharides are bonded through (1 → 3)-d-glucopyranose and (1 → 4)-D-glucopyranuronic acid linkages (Pramanik & Islam, 1998). Their subsequent work emphasized the extraction and purification of polysaccharides from *Moringa* pods using hot water extraction and gel permeation chromatography. This technique determined a molecular weight of 70 kDa, and α-d-glucopyranose was the primary monosaccharide linked through 1 → 4 glycosidic linkages (Mondal et al., 2004). Apart from this, this group isolated and purified a heteropolysaccharide of molecular weight 1.96 × 10^2^ kDa from the pods and identified significant monosaccharides. This method allowed the same team to isolate and purify a heteropolysaccharide of molecular mass 1.96 × 10^2^ kDa from *Moringa* pods. The most abundant monosaccharides are 6-O-Me-D-galactose, D-galacturonic acid, D-galactose, L-arabinose, and L-rhamnose in 1:1:1:1:1 M ratio. The glycosidic linkages involved were (1 → 2)-L-arabinopyranose, (1 → 2)-linked d-galactopyranose, and (1 → 2,3)-linked D- galactopyranose (Roy et al., 2007).

**Table 3:** Types of polysaccharides in the different parts of *Moringa oleifera* Lam.

Leaf	Seed	Flower	Root	Gum	Pod	References
Xylose, mannose, glucose, galactose, arabinose, galacturonic acid, rhamnose	D-galactose, L-rhamnose, L- arabinose, mannose, glucose, uronic acid	d-glucose, D-galactose, D-glucuronic acid	Rhamnose, xylose, arabinose, fructose, mannose, galactose	Arabinose, galactose, rhamnose, galacturonic acid	d-glucose, D-galactose, D-glucuronic acid, L-rhamnose, L- arabinose	Sharma et al., 2022; Yang et al., 2022

Recently, several methods were employed to fractionate a novel type of arabinogalactan polysaccharide designated as F1 with a molecular weight of 190 kDa from the roots of *Moringa* gum (Raja et al., 2016). The total polysaccharide extract and the water-eluted polysaccharide fraction (F1) were reportedly acquired through anion exchange chromatography. HPSEC found the molecular mass of the purified F1 polysaccharide to be 190 kDa. GC–MS and TLC analysis showed that F1 polysaccharide is mainly arabinose with 64 % content, followed by galactose (25 %), xylose (4 %), glucuronic acid (4 %), and rhamnose (3 %). The backbone chain of the oligosaccharide consists of 1 → 6, 1 → 3, and 6-β-galactopyranose linkages. Further study showed that F1 polysaccharide is soluble in water, does not affect the existence of complexes with β-lactoglobulin and contains antioxidant properties. β-lactoglobulin is widely used in food products, especially whey protein, due to its properties, such as foaming, emulsification, and gelation. Interaction between F1 and β-lactoglobulin may provide clues for designing a new functional structure (Raja et al., 2016). Non-starch polysaccharides were isolated from the defatted *Moringa* seed flour using hot and cold-water extraction methods followed by purification through gas-liquid chromatography (Anudeep & Radha, 2018). The investigation showed that cold-fraction polysaccharides contained only 18 % galactose, 8.9 % rhamnose, 3.1 % glucose, and 1.08 % arabinose. Instead, hot-water soluble polysaccharides contained 44.7 % arabinose, 13.1 % galactose, 9.42 % glucose, 2.8 % xylose, and 2.06 % mannose, as shown in Table 3. Recently, a novel polysaccharide named MRP-1 was purified from the roots of *Moringa* in China (Cui et al., 2019). The polysaccharides from the roots were extracted through hot-water extraction. The final supernatant sample was diluted and centrifuged upon precipitation with ethanol, and then purification was carried out using a DEAE-Sepharose fast flow column (Cui et al., 2019). The results showed that MRP-1 had a molar ratio of various monosaccharides, comprising rhamnose, arabinose, fructose, xylose, mannose, and galactose in the following ratios: 1.5:2.0:3.1:6.0:5.3:1.1.

## 3 *Moringa oleifera* derived-metallic nanoparticles (MNPs)

This paper reports on the use of *M. oleifera* in three independent stages for the biosynthesis of metallic nanoparticles: (a) induction phase, wherein metal ore gets reduced to form certain ions of pure iron with the specific basis for the seeding metallic atoms; (b) growth phase, wherein nanoparticle seeds aggregate and trigger spontaneous assembly and further development into large particles. In the final stage of nanoparticle preparation, the particles assume the most favourable energy shape, which significantly depends on the stabilizing activities of *Moringa oleifera* extract (Perumalsamy et al., 2024). For instance, though nanotriangular shapes possess very high surface energies, they are not relatively stable. Since the nano triangular structures cannot stabilize some particular extracts, the nanoparticles transform into more stable configurations of truncated triangles to minimize Gibbs free energy (Perumalsamy et al., 2024). The amine groups in proteins and other secondary metabolites, besides carboxyl and hydroxyl groups of the amino acids and polyphenols present in *Moringa oleifera*, in addition to carboxyl groups in organic acids and hydroxyl groups in polysaccharides, are involved in chelating the metal ions. This method is used to synthesize MNPs due to functional groups in the *Moringa oleifera* phytoextract secondary metabolites (Perumalsamy et al., 2024). Table 4 describes the detailed synthesis of various green MNPs prepared from *Moringa oleifera* biomass and discusses their role in health and environmental applications.

**Table 4:** Type of MNPs synthesized from different parts of *Moringa oleifera* Lam., with their applications.

Type of plant part used	Type of MNPs synthesized	Size (nm)	Shape	Applications	References
Seed	Copper	7.3	NS	It was applied in this study as a high-performance efficient adsorbent to remove several kinds of pollutants such as hexavalent chromium [Cr(VI)] up to 38.6 mg/g, cationic dyes (rhodamine B) and anionic ones (congo red), titan yellow or methyl orange less than within just 10 mins time; The extract has a potent antimicrobial action against *Staphylococcus aureus*, *Enterococcus faecalis*, *Escherichia coli*, *Klebsiella pneumoniae* and *Candida albicans*.	Alsamhary, 2023
Leaf	Copper	21	Spherical	The material presents significant results towards cytotoxicity on the MCF7 (human breast cancer cell line) and A549 (adenocarcinomic human alveolar basal epithelial cells) cancer cell lines; Degrades malachite green and titan yellow by 84.95 % &79.03 %, respectively	Alhamdi et al., 2024
Leaf	Copper oxide	3–5	Spherical	It also shows antifungal action against *Aspergillus flavus*	Prajapati et al., 2023
Flower	Gold	3–5	Spherical	Catalyze the rapid reduction of 4-nitrophenol to 4-aminophenol	Anand et al., 2015
Leaf	Gold	10–20	Spherical	This material has shown to be cytotoxic in the A549 cell line by apoptosis	Tiloke et al., 2016
Seed	Iron	2.6–6.2	Spherical	85 % removal of nitrate ions (NO^3−^) from ground and surface water; Bactericidal against *Escherichia coli*	Katata-Seru et al., 2018
Pod, and Leaf	Iron oxide	45	Irregular	Bactericidal against *Escherichia coli* and *Bacillus subtilis*; High thermal conductivity	Jegadeesan et al., 2019
Leaf	Iron oxide	15	Rod	It has antibacterial activity against different bacteria such as *Escherichia coli*, *Pseudomonas aeruginosa*, *Staphylococcus aureus*, *Shigella*, *Salmonella typhi*, *Pasteurella*	Aisida et al., 2020
Flower	Silver	8	Spherical	Effective against *Klebsiella pneumonia* and *Staphylococcus aureus*; Highly sensitive to detect copper ions	Bindhu et al., 2020
Leaf	Silver	9 and 11	Spherical	It exhibits bactericidal efficiency against *Enterococcus faecalis*, *Escherichia coli*, *Klebsiella pneumonia*, *Pseudomonas aeruginosa*, and *Staphylococcus aureus* and antifungal *Candida albicans*, *Candida krusei*, and *Candida parapsilosis*	Moodley et al., 2018
Leaf	Silver	109	Spherical, and Polygonal	Displays good antileishmanial activity against *Leishmania* major	El-khadragy et al., 2018
Leaf	Silver	Less than 100	Spherical	It exhibits potent cytotoxicity in Kasumi-1(myeloblast) cells through apoptosis	Khor et al., 2020
Seed	Silver	4	Spherical	Significant antibacterial activities against *Staphylococcus aureus*, *Escherichia coli*, *Salmonella enterica typhimurium* and *Pseudomonas aeruginosa*; Outstanding photocatalytic activity for the photodegradation of methylene blue (MB), orange red and 4-nitrophenol; Simultaneously adsorbing lead (Pb^2+^) ions from water.	Mehwish et al., 2021
Leaf	Silver	50–60	Spherical, and Semispherical	It displays strong anti-microbial effects against *Staphylococcus aureus* and *Candida glabrata*; Cytotoxic activities against malignant melanoma cell line (A375)	Mohammed & Hawar, 2022
Leaf	Silver	5–50	Spherical	It showed significant antibacterial activity to various bacterial strains such as *Escherichia coli, Serratia marcescens*, and *Bacillus subtilis*	Abdel-Rahman et al., 2022
Stem	Silver	3–70	Spherical	Effective against against *Escherichia coli*, *Klebsiella cloacae*, and *Staphylococcus epidermidis, Spodoptera littoralis*; Cytotoxic activity against HCT-116 (human colorectal carcinoma cell line), HepG-2 (human liver cancer cell line), MCF-7 (human breast cancer cell line); Reduced 2, 4-di-nitrophenol to 2, 4-diaminophenol	Arrieta et al., 2017
Gum	Silver	50	NS	Effective against methicillin-resistant *Staphylococcus aureus* (MRSA), *Escherichia coli*, and *Staphylococcus aureus*	Irfan et al., 2021
Gum	Zinc oxide	60	NS	Antibacterial activity against *Escherichia coli*, *Staphylococcus aureus*, and methicillin-resistant *Staphylococcus aureus* (MRSA)	Irfan et al., 2021
Leaf	Zinc oxide	52	Hexagonal	Nearly 96 % of titan yellow dye is completely degraded	Pal et al., 2018
Leaf	Zinc oxide	50	Flower like	Significant antibacterial activity against *Escherichia coli bacteria*, as well as *Staphylococcus aureus*	Abdel et al., 2021

NS: Not specified.

Jaswanth et al. (2021) synthesized copper nanoparticles (CuNPs) in situ on nanocomposite fabrics (NCFs) through an eco-friendly green synthesis method using *Moringa oleifera* leaf extract as a reagent. Thermogravimetric analysis showed that the NCFs were within the thermal stability limits up to a temperature of 404 °C. The NCFs showed good tensile strength and strong antibacterial action against Klebsiella pneumonia and *Staphylococcus aureus* at greater load*s*. Moreover, the CuNPs synthesized using a hydroalcoholic extract from the leaves of *M. oleifera* were highly potent with their antimicrobial activity against bacteria *Escherichia coli*, *K. pneumoniae*, *S. aureus*, and *Enterococcus faecalis* with MICs of 500, 500, 500, and 250 μg/ml, respectively (Das et al., 2020). The antifungal activity of CuNPs was also substantially significant towards Aspergillus niger, *A. flavus*, *Candida albicans*, and *C. glabrata*, 125, 125, 62.5, and 31.2 μg/ml, respectively. Kiran et al. (2021) reported that the gold nanoparticles (AuNPs) synthesized from *M. oleifera* leaves independently exhibited significant antidiabetic activity concentration. Moreover, MO-AuNPs showed extreme anticancer activity, and an IC_50_ value of 67.92 μg/ml was recorded in cytotoxicity assays against human breast cancer cell lines (MCF-7). Choudhary et al. (2023) reported that iron nanoparticles were synthesized from *M. oleifera.* Concentrations below those conventional for antibacterial treatments were required for *M. oleifera* leaves to show significant antibacterial activity. Silveira et al. (2018) reported that iron-oxide nanoparticles (NPsFeO) obtained from *M. oleifera* leaves exhibited effective fluoride ion adsorption with an equilibrium time of 40 min compared to that required by BGAC (granulated bone charcoal), which was 90 min. Adsorption capacities in both cases were 1.40 mg/g and 1.20 mg/g, respectively. More notably, silver nanoparticles (AgNPs) prepared from *Moringa oleifera* seed cake had been reported to possess significant antibacterial activity against *E. coli* BL21(DE3) (Coelho et al., 2023). Younas et al. (2023) have reported that AgNPs prepared from *M. oleifera* leaves showed increased antibacterial activity against *K. pneumoniae*, *E. coli*, *Acinetobacter baumannii*, and *Pseudomonas aeruginosa*. Besides, AgNPs derived from *Moringa oleifera* leaves were found to possess cytotoxicity against MCF-7 cell lines, and their IC_50_ value was 5 μg/ml (Alkan et al., 2022). Ramzan et al. (2022) investigated the synergistic effect of zinc oxide nanoparticles (MZnONPs) fabricated from *Moringa oleifera* leaves and leaf extract to alleviate cadmium (Cd) stress in linseed (*Linum usitatissimum* L.). Their results revealed that MZnONPs application to Cd-stressed linseed plants drastically improved the growth of the plant and raised the levels of antioxidant enzymes. Furthermore, the addition of MZnONPs to Cd-stressed seedlings enhanced remarkably the activities of superoxide dismutase (SOD), peroxidase (POD), catalase (CAT), and ascorbate peroxidase (APX). The MZnONPs also lowered the contents of malondialdehyde (MDA) and hydrogen peroxide (H_2_O_2_) in Cd-treated linseed plants. In addition, the nanoparticles inhibited electrolyte leakage (EL) in the leaves and roots of linseed plants under Cd stress (Ramzan et al., 2022).

It has been demonstrated that plant material decompositions of metal ions were much higher than bacteria and fungi since selective extracts from plants could be used as coordination agents (Kumar et al., 2024). Consequently, the fact that there are specific coordination properties associated with nanoparticles produced from plant extracts can establish that plant-based synthesis may offer a promising strategic approach towards future large-scale applications of MNPs. Aggregated MNPs, in this case, are produced from plant resources is a greener, less costly, and more sustainable way. This process further ensures that the generation of harmful waste is at an all-time low while ensuring the manufacturing process is clean for the environment. Dikshit et al. (2021) have illustrated various difficulties in synthesising MNPs through green processes. Generally, by extensive optimization experiments on process parameters-picking speeds of rotation and temperature, pH values-and reactants such as plant materials-type and quantity-types of solvents and solvents' volumes, MNPs that appear consistent in shape and size must be produced.

Additionally, with time, further optimization of several reaction parameters is critical to maximize the yield and stability of MNPs while minimizing the reaction time. Numerous in vitro studies have demonstrated the antimicrobial and antioxidant potential of nanoparticles that have been synthesized using *Moringa* extract. However, in sufficient in vivo data poses considerable challenges for translating these findings into practical applications (Perumalsamy et al., 2024). Even though, there is a significant gap in understanding the pharmacokinetics and the specific mechanisms responsible for the cytotoxicity of these nanoparticles (Perumalsamy et al., 2024). Further research is required to ascertain the optimal size and morphology of these nanoparticles to be ensured of desired therapeutic outcomes. Addressing these gaps is critical for the development of *Moringa*-based nanoparticles in biomedical and therapeutic applications.

## 4 *Moringa oleifera*-derived biochar

Biochar, obtained through pyrolysis of different types of biomasses, has a dual purpose. It can be used as an energy source and a promising adsorbent (Cao et al., 2019; Tomczyk et al., 2020). Generally, it is produced from low-cost feedstocks that are easily accessible, including animal residues and plant-based products like bagasse, agricultural rains, seed husks, seeds, and organic wastes (Hoslett et al., 2019; Tan et al., 2015). Biochar essentially contains high carbon amounts, with small levels of nitrogen and hydrogen and trace elements such as calcium, magnesium, potassium, and sodium. The biochar is generally characterized by a large number of surface functional groups in combination with a highly specific surface area (da Silva et al., 2024). The type of biomass used in its preparation significantly impacts the key physicochemical properties, which are the specific surface area and the composition of the surface functional groups (da Silva et al., 2024). Biochar was synthesized from *Moringa oleifera* seed shells through pyrolysis of the biomass to remove metronidazole from aqueous solutions, according to da Silva et al. (2024). Activation using phosphoric acid (H_3_PO_4_) enhanced the capacity up to 18 mg/g, whereas potassium hydroxide (KOH) activation significantly enhanced the capacity up to 366.4 mg/g. Pyrolysis was performed by Prasad et al. (2022) using wood from calcium-pretreated *M. oleifera*. The biochar produced has high capacities as an adsorbent, resulting in 37.2 mg/g removal of arsenate and 33.4 mg/g of arsenite from aqueous solutions. Raw and biochar derived from *M. oleifera* by Tavengwa et al. (2016) reported that biosorbents containing *M. oleifera* seed powder were successfully used to remove nitrobenzene (NB) from aqueous solutions. The concentration of NB was estimated using an HPLC-UV detector with a limit of detection of (11.54 μg/l) and a limit of quantitation of (38.46 μg/l). Li et al. (2022) synthesized biochar from *Moringa oleifera* seed shells through sonication-assisted impregnation and subsequent pyrolysis. Then, the resulting biochar was activated by Fe_3_O_4_ to fabricate Fe_3_O_4_-MOS, which was used to remove MB from water using aqueous solutions. The primary adsorption mechanisms in the Fe_3_O_4_-MOS case were determined as pore adsorption, electrostatic interaction, hydrogen bonding, and π-π interaction. Even after five cycles of adsorption-desorption, Fe_3_O_4_-MOS maintained a high removal efficiency for MB, which was higher than 90 %. In addition, surface-modified biochars from *M. oleifera* leaves were synthesized and used to remove MO dye from aqueous solutions (Islam et al., 2022). H_3_PO_4_ was used at different ratios to optimize the number of functional groups on the surface, the pore structure, and the stability of the biochar. The maximum adsorption capacity was obtained by producing biochar at 500 °C with an impregnation ratio of 1.5 with 175 mg of MO dye/g of activated biochar. Mishra & Mohanty, 2023 refer that pyrolysis-derived biochar usually suffers from insufficient surface functional groups and porosity. Thus, functionalization is necessary to make it pragmatic. Diverse physicochemical modifications are undertaken to enhance surface area and porosity that qualitatively improves its applications in bio-composites, catalysis, energy storage, environmental remediation, nanotubes, and supercapacitors (Kumar et al., 2024). Study conducted by Roy et al. (2022) highlighted the efficiency of biochar derived from *M. oleifera* depends on the specific application and use of the composite.

## 5 *Moringa oleifera* derived bio-adsorbent

The search for alternative cheap techniques that might remove harmful pollutants from wastewater has recently emphasized biosorption (Ngeno et al., 2024). This technology is characterized by its flexibility and cost-effectiveness, incurring minimal energy requirements, while biosorbents could be easily regenerated. This technique utilizes the ability of agricultural waste and other biological materials to adsorb contaminants efficiently (Ahalya et al., 2006; Jabar & Odusote, 2024). Carbonaceous porous adsorbents such as activated carbon, carbon nanotubes, charcoal, and powdered materials can be used with or without chemical and thermal modifications (Ngeno et al., 2022). However, the biosorbent dosage, contact time, initial concentration, pH, and temperature determine the removal percentage in the biosorption process. The key properties associated with biosorbents include morphology, surface pH, density, and type of functional groups and conditions under which they are activated (Chimi et al., 2023; Jabar et al., 2025). Also, the adsorbate properties such as dissociation behaviour, hydrophobicity, kinetic diameter, and polarity play a crucial role as factors that dramatically influence the efficiency of biosorption (Shikuku et al., 2018). Hence, excellent biosorbent is characterized by cost-effectiveness, a wide range of contaminant adsorption, high biosorption capacity, and rapid kinetics (Ngeno et al., 2022). Table 5 illustrates the diversities of the applications of bio-adsorbents obtained from different parts of *M. oleifera*.

**Table 5:** Bio-adsorbent derived from the different parts of *Moringa oleifera* Lam.

Type of plant part used	Drying temperature and time	Applications	References
Seed	300°, 1 h	A low-cost adsorbent for the continuous adsorption of the herbicide atrazine in a packed-bed column was provided	Bergamasco et al., 2023
Seed	600°, 2 h	Over 85 % elimination within BOD and phosphate from wastewater	Hendrasarie & Maria, 2021
Seed husk	573 K, 1 h	It shows the maximum adsorption capacity for atrazine as 10.3 mg/g	Cusioli et al., 2021
Seed husk and pulp	60°, 48 h	Biosorption of acid blue 9 synthetic dye by seed husk and pulp is 329.55 mg/g,694.2 mg/g respectively	Escobar et al., 2021
Seed coat	105°, NS	Maintaining the optimum conditions of a 321 K temperature and contact time at 90 min, more than 90 % removal was obtained for Congo red dye from aqueous solution using from experimental study	Jabar et al., 2020
Leaf	70°, 20 h	Maximum biosorption of (Pb) ions (45.83 mg/g) was achieved with (0.15 g/100 ml) adsorbent dosage while highest removal (98.6 %) was obtained at adsorbent biomass 1.0 g/100 ml and pH 6	Imran et al., 2019
Pod	45°, 48 h	A higher removal rate was achieved when chromium (Cr (VI)) and naphthol blue black (NBB) dye were removed in the topsoil layer, with a total of 91.8 % for Cr (VI), fine fraction, mixed fraction at root zone depth, and coarse fractions having efficiencies of 74.9 %,52.6 % respectively; In case of NBB the removal efficiency for the same fractions was 97.5, 33.6, 18.9 %, respectively	Shirani et al., 2018
Leaf	NS	The material efficiently removes cadmium (II) in water	Ali et al., 2015

NS: Not specified; BOD: Biochemical oxygen demand.

Alghamdi et al. (2024) removed manganese (Mn) from an aqueous solution using three particle sizes of *M. oleifera* seeds and two acidic pH levels. Optimum adsorption conditions were realized after 2 h of contact time with the combination of pH 3 and 250 μm size with dosages of 0.2 g at room temperature, proving to be over 92 % in metal removal efficiency. Ngeno et al. (2024) synthesized powder, fat-free *M. oleifera* seed biomass (MOSB), activated with H_3_PO_4_, as a low-cost biosorbent for the removal of the progesterone (PGT) hormone from synthetic wastewater. The best conditions of PGT biosorption were determined to be: contact time of 86.8 min, a temperature of 298 K, an adsorbent dosage of 0.1 g, and an adsorbate concentration of 500 μg/l. Moreover, PGT biosorption from aqueous solutions was pH-independent, with a biosorption mechanism and a predominant physisorption component that excluded electrostatic interactions. Damásio et al. (2022) demonstrated that seeds of *M. oleifera* can be an effective bio-adsorbent in obtaining 100 % removal of diclofenac (DCF) from contaminated drinking water samples. The removal batch process of DCF was carried out under optimal conditions when the extraction time was 30 min, and the seed mass was 2.0 g, pH 5.0, and 25 ml of 10.0 mg/l DCF. Jayan et al. (2021) investigated the removal of lead (Pb (II)) and zinc (Zn (II)) ions from aqueous solutions using the *M. oleifera* leaves (MOLs) of the Periyakulam-2 variety. Different isotherms and kinetic models were used to analyze the adsorption data. It was seen that the Langmuir isotherm model provided the best fit with maximum adsorbing capacities for lead and zinc at 51.71 mg/g and 38.50 mg/g, respectively. Kinetic studies for lead and zinc adsorption confirmed that the process followed the pseudo-second-order model. Chemical functionalization of the bio-absorbent enhances its selectivity in adsorbing a specific molecule (Kumar et al., 2024). Raw *M. oleifera* serves as a cost-effective material with high values of maximum adsorption capacity. However, numerous researchers have explored different adsorbent modification approaches. The modification approaches include activated carbon preparation, chemical functionalization (employing acidic or basic treatments), synthesis of composites as well as organic reactions like acryloylation and esterification. These modifications aim to increase the adsorption efficacy of *Moringa* based materials for diverse applications (Gomes et al., 2022).

## 6 *Moringa oleifera*-derived biodiesel

**Fig. 2:**
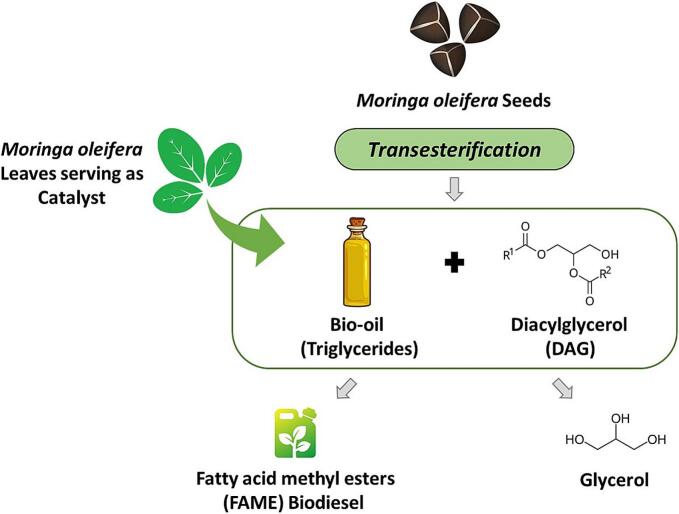
Schematic presentation of biodiesel production using *M. oleifera* as a catalyst and source of FAME.

Biodiesel is a renewable fuel primarily derived from vegetable oils, animal fats, and biomass. B100 is a type of biodiesel composed of monoalkyl esters of long-chain fatty acids. It must meet the quality standards specified in the ASTM D6751 (Azad et al., 2015). Biodiesel consists of fatty acid methyl esters (FAME), which are produced from various vegetable oils, including corn oil, cottonseed oil, jatropha oil, palm oil, peanut oil, rapeseed oil, soybean oil, and sunflower oil. It can also be sourced from alternative materials such as animal fats, greases, microalgae, and waste cooking oil (Azeem et al., 2016; Krisnangkura, 1986). Various physicochemical methods have been applied for biodiesel production, including esterification, pyrolysis, supercritical fluid processes, and transesterification (Kumar et al., 2024). Currently, the majority of biodiesels are produced from edible oil resources, which has caused an increase in food prices and added pressure on land resources (Azad et al., 2015). This has led to a change towards using biodegradable, safer, non-edible, and less polluting resources for biodiesel production (Azad et al., 2015). The mature seeds of *M. oleifera* produce 38–40 % colourless and odourless vegetable oil (Azad et al., 2015). However, toxic compounds have been found in this oil, making it incapable of human consumption. Therefore, it is categorized as non-edible (Niju et al., 2019). The direct application of *M. oleifera* oil for transesterification is dominated by soap formation due to its high acid value (Niju et al., 2019). The two-step approach is a dominant biodiesel production method, including esterification followed by transesterification. *Trans*-Esterification is a reaction whereby triglycerides in feedstocks react with alcohol to yield glycerol and fatty acid alkyl ester as by-products of the process (Nayak & Vyas, 2022). This process converts *M. oleifera* oil into biodiesel. Such interest from the biofuel industry is unprecedented since *M. oleifera* oil has the characteristic attributes, and therefore, it remains one of the prospective feedstocks in biodiesel production (Niju et al., 2019). Transesterification requires catalysts to achieve a somewhat high biodiesel yield at lower temperatures. Alkaline homogeneous catalysts of NaOH and KOH and acids H_2_SO_4_, HCl, and H_3_PO_4_ are usually used and preferred because they possess excellent catalytic activity, are readily available, and cost less compared to other base catalysts (Aleman-Ramirez et al., 2021). Although highly active and efficient, such catalysts are corrosive and generate vast amounts of waste during backwashing. In addition, the energy-intensive separation techniques necessary for the recovery of the reaction products cannot always be applied economically to recycle the recovered catalysts. A new class of catalysts based on biomass residues has increasingly been developed in the search for sustainable solutions within green chemistry. These catalysts have delivered an eco-friendly approach and economic feasibility due to the reuse of the waste material for low-cost fuel production (Tang et al., 2018). The biomass-derived catalysts contain some environment-friendly attributes, including biodegradable properties and nontoxicity with easy structure enhancement of pores and surface area for performance modification (Farooq et al., 2018). A high alkaline mineral content in *M. oleifera* leaves makes them a sustainable and competitive precursor for catalyst development (Fig. 2).

Soudagar et al. (2021) produced biodiesel from *Moringa oleifera* oil through transesterification using 1 % wt KOH as an alkaline catalyst. The studies resulted in lowering brake power, brake-specific fuel consumption (BSFC), and CO emissions by 7.74 %, 7.61 %, and 7.7 %, respectively. Aleman-Ramirez et al. (2021) formulated a heterogeneous catalyst using *Moringa oleifera* leaves as a green alternative for biodiesel production. The catalyst calcination process was performed at 500 °C for 2 h to produce inorganic carbonate minerals like calcite, dolomite, and fairchildite (K_2_Ca(CO_3_)_2_). A mixture of the final catalyst with oil and methanol could provide for in situ biodiesel synthesis. All the minerals formed during calcination were essential for the transesterification reaction. The research indicated that under optimal operating conditions using a methanol-to-oil molar ratio of 6:1, catalyst loading of 6 %wt, and a reaction temperature of 65 °C for 120 min, FAME yields reached an impressive 86.7 %. The researchers also proved that the catalyst was recyclable in up to three successive batches without losing any significant amount of efficiency. Teoh et al. (2019) obtained a considerable reduction of carbon monoxide (CO), particulate matter (PM), and smoke emissions without any alteration of the engine using biodiesel from *M. oleifera* or its blends in a multi-cylinder high-pressure common-rail diesel engine; nitrogen oxide (Nox) emission was not altered. According to Valenga et al. (2019), using *M. oleifera* leaves as an antioxidant increases the storage time of soya oil biodiesel to about 6.5 months, thus preventing degradation through oxidative reactions. Fernandes et al. (2015) tested a focus on using ethanolic extracts from *M. oleifera* leaves as additives for increased induction period (PI) values for biodiesels, which improves their stability. The IP values of soybean biodiesel were significantly increased by employing the 98 % ethanol extract from the *Moringa oleifera* leaves compared to tert-butylhydroquinone. Consequently, it proved that soybean biodiesel required 100 μ/g of the extract to elongate the IP up to 10.3 h from only 3.8 h documented using other synthetic antioxidants. This confirmed an improvement better than that of the synthetic antioxidant tertbutyl hydroquinone. According to Azad et al. (2015), the biofuel derived from *Moringa oleifera* had relatively inferior performance and marginally higher NOx emissions than fossil fuels in Australia.

## 7 Improved utilization of different biomasses of *M. oleifera* as food

**Fig. 3:**
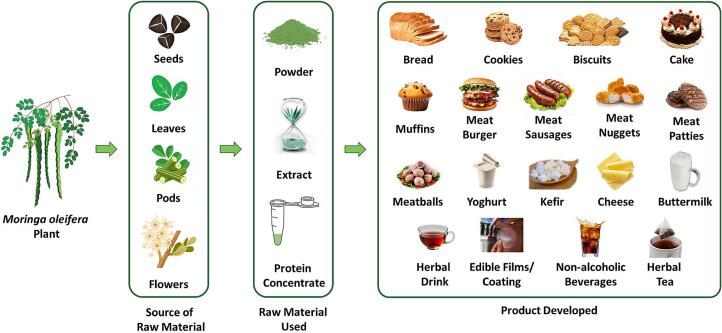
Various functional food production using *M. oleifera* different biomasses.

Capozzi et al. (2022) highlighted how technological advancement has made it easier to develop more effective natural additives and nutritional ingredients from the food industry by-products. Through the application of the circular economy model, new sources of nutrition have developed, with quality ranging from equivalent to surpassing traditional alternatives, while also providing for the increasing need for healthy foods and constant drives towards sustainability in production (Capozzi, 2022). From this perspective, food fortification offers an appropriate solution to eliminate nutritional deficiencies (Naozuka, 2018). As such, people reliant on processed foods are likely to adopt healthier fortified alternatives that curb deficiencies in critical nutrients, including vitamins (Kumar et al., 2024). One of the ways *Moringa oleifera* leaves are used is in developing countries, which act as a source of vitamin A, preventing vitamin A deficiencies (Nambiar & Seshadri, 2001). The food industry can improve traditional products through sustainable means by including several parts of the *Moringa* plant (Trigo et al., 2023). In addition, various *Moringa* biomass has been used in foods, significantly affecting the colour, appearance, flavour, and texture. Fig. 3 represents some food products prepared using different parts of *Moringa oleifera*, including seeds, leaves, and flowers.

### 7.1 Cereal-based food

Cereal-based foods, such as cookies, cakes, bread, biscuits, muffins, and cupcakes, are now staples in our daily diet, providing a high carbohydrate and protein source. However, these products lack essential micronutrients such as minerals, vitamins, phytochemicals, and fibre. With the increased consumer demand for healthier food options, fortifying functional ingredients in staple food items has been another trend (Kumar et al., 2024). Among the various functional ingredients, flowers, seeds, and leaves of *M. oleifera* are incorporated into cereal-based food products as extracts, protein concentrates, and powders, as highlighted in Table 6**.**

**Table 6:** Cereal-based food developed using different parts of *Moringa oleifera* Lam.

Developed product	Plant part used and its form	Quantity added	Product quality	References
Bread	Leaf powder	5, and 10 %	It improves the physical properties of dough and bread in terms of protein content, water holding capacity as well as imparting fruity aroma; It is high in fibre with inulin	Nudel et al., 2023
	Leaf powder	5 %	Fortified bread had a higher total phenolic content (TPC) and antioxidant properties than in non-fortified bread	Ferreira et al., 2023
	Powder	0, 1.2, 2.5, 5, and 10 %	Photoacoustic signal amplitude values of bread raised from (37–90 %) when moringa powder concentration rised from (1.25–10 %), at 300 nm wavelength; The sanitary quality of bread mixed with a 2.5 % of moringa was higher than ones obtained for different concentrations; It slowed down its textural changes of bread (hardness, elasticity, cohesiveness, resilience, and chewiness)	Hernandez-Aguilar et al., 2021
	Leaf powder	2.5, 5, 7.5, and 10 %	Addition of leaf powder greater than 2.5 % reduced the specific volume of bread; The hardness and chewiness of bread slightly reduced with 2.5 and 10 % addition, springiness remained unaffected; For sensory evaluation, gluten-free bread was obtained for control bread and bread with 2.5 % the latter being more acceptable	Bourekoua et al., 2018
	Seed flour	0, 2.5, 5, and 7.5 %	Seed flour addition significantly (*p* < 0.05) increased the protein (13.8–15.3 %), fat (1.2–1.5 %), ash (1.5–1.6 %) and fibre content (0.13–0.18 %) of the bread samples, while the moisture (7.8–7.6 %), carbohydrates content (75.4–72.8 %) and pH (8.05–7.8 %) of the bread decreased; 5 % fortification level was acceptable to consumer	Eke et al., 2022
Biscuit	Leaf powder	5 %	The fortified biscuit had better TPC and antioxidant activity	Ferreira et al., 2023
	Flower powder, and leaf powder	11:4, 11.7:3.2, 12.5:2.5, 13.2:1.7, and 14:1	The calcium content (115.73 mg/100 g) was higher in biscuits with a higher percentage of the flower and leaf ratio (11:4) while TPC was less	Yadav et al., 2022
	Seed protein concentrate	2, 4, and 6 %	Progressive increase in crude protein content owing to the supplementation; The overall quality acceptability did not show any statistically significant difference among the biscuit samples; The microbiological result were within the safe range for baked products	Aderinola et al., 2020
	Leaf powder	2, and 4 %	The biscuits made from both percentages was acceptable by the panellists	Pathak & Jain, 2021
Cake	Leaf powder	1, 1.5, and 2 %	Supplementation with 1.5 % provided the enriched nutritional quality and contributed to the improvement of food and nutritional security of the vulnerable populations	Roni et al., 2021
	Leaf powder	0, 2, 4, 6, 8, and 10 g	The cake sample with 4 g *Moringa* addition was the most preferred in terms of colour, taste, aroma and general acceptability	Kolawole et al., 2013
Cookies	Leaf powder	2.5, 5, and 10 %	Thickness were significantly (p < 0.05) reduced while diameter and spread ratio of the cookies increased; significant (p < 0.05) enhancement in the bioactive compound, antioxidant and inhibitory properties; The sensory evaluation revealed cookies having 2.5 % powder were more acceptable	Fapetu et al., 2022
	Leaf powder, and seed powder	2.5, 5, and 7.5 %	As the substitution level increased, the volume and specific volume of cookies gradually decreased in comparison with control sample; Substitution of wheat flour (72 % extraction rate) with moringa leaf and seed powders decreased the cookie diameter in all cases but increased its thickness	Rabie et al., 2020
	Leaf powder	0, 5, 10, and 15 %	The protein, dietary fibre and mineral content of cookies were found to increase upon addition with different levels of leaf powder; Sensory evaluation indicated that cookies containing <10 % moringa leaf powder were significantly different from the control in terms of quality, aroma, and taste	Mouminah, 2015
Muffins	Leaf powder	0, 3, 6, 9, and 12 %	Increased the mineral content	Rismaya et al., 2023
	Leaf powder	8, 10, and 12 %	Addition of 12 % powder was most preferred	Srinivasamurthy et al., 2017
Chinchin	Leaf powder	0, 1, and 5 %	5 % leaf powder resulted in significantly (p < 0.05) higher phenolic content, antioxidant activity, protein content, ash and oil uptake	Famakinwa et al., 2023

Agba et al. (2024) investigated the incorporation of decolourised *M. oleifera* leaf powder (D-MOLP) into cookies to enhance their nutritional value beyond eliminating consumers' resistance to using the green colour the superfood imparts. The study established that neither a 2.5 % nor a 7.5 % addition of D-MOLP significantly impacted the functionality properties of flour or water activity values of the baked cookies. The colour was slightly different in the cookies. *Moringa*-enriched cookies had elevated phenolic and protein content and higher antioxidant activity with a better spread ratio and increased in-vitro protein digestibility compared to control cookies. Cookies analysis showed the presence of the following phenolic acids: fumaric, ferulic, and chlorogenic. Antinutrients are reduced, making D-MOLP cookies of better quality and higher nutrient content. Endah et al. (2024) added *Moringa* leaf flour to muffins, resulting in higher ash, carbohydrates, fat, moisture, and protein content. However, the incorporation affected the colour, taste, aroma, and overall quality acceptance of sensory attributes. It also decreased the baking time and moisture content and enhanced the sensory acceptance of colour while improving the texture of the muffins, making them more acceptable in general. Flatbread or Chapati was baked using 5 % and 10 % *Moringa* leaf powder, by which the sensory characteristics were enhanced, with overall acceptance scores being 6.90 and 6.20, respectively (Mushtaq et al., 2018).

Khan et al. (2023) studied the possible use of 5 % *Moringa* leaf powder (MLP) in bread. The results indicated that the bread incorporated with MLP was quite acceptable. Additionally, the study showed prominent nutritional, mineral, and antioxidant improvement in the bread after adding MLPs. It would, therefore, mean that MLP could be a good tool for improving nutrition status in developing and underdeveloped countries. Debittered *M. oleifera* is one processing form of the plant that removes bitterness. The rheological and physicochemical properties, sensory attributes, microstructure, and nutritional characteristics of cookies fortified with debittered *M. oleifera* seed flour (DDMF) at different levels of incorporation: 25 %, 50 %, 75 %, and 100 % were discussed by Agrawal et al. (2022). Findings indicate that a progressive increase of DDMF from 0 % to 100 % increased the water absorption content from 59.5 % to 77 %, raised the hardness of cookie dough from 89.2 N to 284.7 N, reduced pasting temperature from 60.2 °C to 30.1 °C, and also reduced the peak viscosity of 696 BU to 9 BU. The sensory evaluation showed that the presence of cookies with 50 % DDMF was highly acceptable, with a clean mouth feel without bitterness. However, DDMF above this threshold made the cookies brittle. The incorporation of 50 % DDMF increased the fatty acid and mineral content and augmented in-vitro protein digestibility.

### 7.2 Meat-based food

Kartikasari et al. (2024) found that supplementation of raw beef patties with *M. oleifera* leaf flour extract (MOLFE) significantly reduced pH levels (*p* < 0.05). In the meantime, there was no variation between treatments regarding hedonic testing (*p* > 0.05). Consequently, consumers accept the addition of 4 % MOLFE in beef patties. Swastike et al. (2024) found that *M. oleifera* leaf extract could be an excellent strength-enhancing additive in beef patties. The maximum level of the *M. oleifera* leaf extract powder (MOLEP) for an application as a strength-enhancing additive to beef patties would be 0.3 %. In addition, including MOLEP in beef patties also prevents it from shrinking while it is being cooked to a certain extent. Rahman et al. (2020) evaluated the anti-oxidative activity of *M. oleifera* leaf extract in goat meat nuggets, compared to 0.1 % beta hydroxyl anisole (BHA). The findings showed that the nuggets containing *M. oleifera* leaf extract were kept fresh during storage, with slight changes in microbiological, physico-chemical, and organoleptic properties after frozen storage at −18 ± 1 °C for 45 days (Table 7). *M. oleifera* leaf extract could be an efficient natural substitute for synthetic antioxidants. Rasak et al. (2021) reported that adding *M. oleifera* leaf powder to beef meatballs reduced cooking loss and enhanced antioxidant power. An inclusion level of 1.5 % *M. oleifera* leaf powder decreased brightness, whereas an addition of 1 % decreased the redness and yellowness values in the colour of the meatball. Additionally, Ibrahim et al. (2022) established that the incorporation of *Moringa* leaf powder into chicken burgers resulted in the retardation of lipid oxidation and peroxide value (PV) during storage of 10 days. The study thus concluded that treatments by *Moringa* effectively slowed down the meat degradation process, hence its potential as a natural preservative to extend the shelf life of chicken burgers.

**Table 7:** Meat-based food developed using different parts of *Moringa oleifera* Lam.

Developed product	Plant part used and its form	Quantity added	Product quality	References
Goat meat nuggets	Pod powder	1.5, and 3 %	Significant differences in lightness and redness of nuggets (p < 0.05) were observed by adding immature moringa powder at a final level of 3 %; Supplementation of moringa pod powder in meat formulations could potentially inhibit lipid oxidation without any change on organoleptic properties	Das et al., 2024
Pork meatballs	Leaf and pod extracts	0.2, 0.4, and 0.8 %	Meatballs prepared with moringa pod extract scored higher sensory attributes compared to those made from leaf extract; A 0.8 % concentration of moringa pod extract significantly delayed lipid oxidation and reduced microbial growth in pork meatballs stored at refrigerated storage	Prasajak et al., 2021
Ground beef	Leaf powder	0.2, 0.4, 0.6, and 0.8 %	The ash, protein, polyphenolic compounds, pH colour significantly increased as an effect of higher content of moringa powder encountered during cold storage meanwhile that moisture, fat, and thiobarbituric acid reactive substances (TBARS) decreased significantly with increased concentration; Moringa leaf powder did not affect the sensory attributes of stored ground beef at level of addition (0.2 % and 0.4 %) used in study	Mashau et al., 2021
Chicken meat nuggets	Flower extract	1, and 2 %	The presence of moringa extract in products significantly decreased the redness, increased the L* value; Texture analysis revealed less hardness, gumminess and chewiness were observed compared to control across all product with 2 % (*w*/w) moringa incorporation; The extract significantly reduced lipid oxidation during storage; Sensory properties of nuggets were not affected	Madane et al., 2019
Chicken patty	Leaf powder	0, 50, and 100 g/kg	A concentration of 50 g/kg moringa leaf powder significantly (*p* ≤ 0.05) retarded lipid peroxidation and extended the shelf-life without jeopardizing sensory attributes of stored chicken patties	Elhadi et al., 2017
Mutton patty	Leaf extract	1, 2, 3, and 5 %	The protein, ash, total phenolic, and total flavonoids contents increased while moisture and fat contents decreased during storage	Mashu et al., 2021b
Chicken sausage	Leaf powder	0.25, 0.5, 0.75, and 1%	Sausages with 0.5 %, 0.75 % and 1 % powder had (p < 0.05) reduced pH values from the 2nd week to the 5th week of storage and significantly (p < 0.05) less total plate count during storage, compared to 0.25 % powder and the control samples; The sensory panel did not detect any difference in products with 0.25 % and 0.5 % powder compared to the control	Jayawardana et al., 2015
Beef burger	Seed flour	0, 2, 4, and 6 %	The cooking characteristics of patties produced from moringa seed flour was beneficially enhanced; The highest thiobarbituric acid values were observed in non-formulated patties after storage; The overall pH of raw patties was decreased during the time and decreased their aerobic bacterial counts significantly (p ≤ 0.05) due to moringa seed flour addition throughout the storage period	Al-Juhaimi et al., 2016

### 7.3 Dairy-based food

Ultrasound-assisted solid-liquid extraction was applied to extract the *M. oleifera* leaf powder. Gomes et al. (2023) studied the physicochemical and biological characteristics of yogurts enriched with this leaf powder. The yogurts supplemented with *M. oleifera* after eight weeks showed no adverse effects, whereas positive and negative controls. In addition, the fortified yogurts possessed significantly greater antioxidant activity compared to the negative control (Table 8). Such findings depict that the potential for *M. oleifera* powder and extract as natural additives in fortifying foods to combat malnutrition could be realized. Sandesh is an Indian sweetmeat widely used within the eastern region of India. It originated in chhana, a form of cheese. An herbal version of Sandesh was developed using cow milk and soy milk, which were fortified with *M. oleifera*. Adding a 3 % concentration of leaf extract decreased the standard plate count (SPC) of yeast and mold in the final product (Giram et al., 2022). In 2015, Salem and his colleagues showed that the inclusion of *M. oleifera* leaf extract (MOLE) at levels of 600, 800, and 1000 ppm into sour cream reduced the total microbiological count (TMC). In addition to that, the sour cream had a typical taste, body, and texture even after long storage periods. Dry leaves of *M. oleifra* (DLMO) was incorporated into labneh cheese, which had excellent true protein digestibility (TD), net protein utilization (NPU), and biological value (BV) (Salem et al., 2013). The organoleptic evaluations proved that the Labneh cheese enriched with DLMO had acceptable qualities throughout the storage period. As Quintanilha et al. (2021) mention, the addition of 2 mg/l ultrafiltered *M. oleifera* seed extract in yogurt reduced the possibility of syneresis, enhanced its firmness, and presented a more uniform and denser microstructure. These characteristics indicate the prospect of introducing *M. oleifera* seed extracts to enhance yogurt's overall quality and consistency.

**Table 8:** Milk-based food developed using different parts of *Moringa oleifera* Lam.

Developed product	Plant part used and its form	Quantity added	Product quality	References
Goat milk kefir	Leaf powder	0, 0.5, 1, 1.5, and 2 %	Acidity, TPC and DPPH activity were increased; Meanwhile the alcohol level was decreased in kefir with supplementation of moringa leaf powder	Wulansari et al., 2021
Yogurt	Leaf extract	1, 3, and 4 %	The addition of moringa extract in milk before fermentation decreased the time required for the complete acidification and produced yogurt with low pH values, therefore increasing bacterial growth during storage; Consequently, rheological features were also improved with increase in TPC and antioxidant capacity of yogurts	Jakopović et al., 2022
Yogurt	Leaf powder	0.5, 1, 1.5, and 2 %	Yogurt fortified with 1 % moringa leaf powder maintained significantly higher scores of sensory attributes such as body, texture flavour, taste and overall acceptability for up to15 days	Saeed et al., 2021
Yogurt	Seed flour	0.1–0.5 %	The addition of 0.1 % and 0.2 % moringa seed flour resulted in the highest contents of Ca, P, K and Fe as well as lower Mg and Zn content with significant bactericidal activity	Dhawi et al., 2020
Soy milk	Leaf extract	0.2, 0.5, and 0.7 g/l	Test results showed that soy milk with a low addition of moringa extract (0.26 g/l) were preferred over higher concentrations; The calcium and iron levels in soy milk were improved at an average by 9 % and by 32 %, with the addition of only 0.26 g/l moringa extract	Rachmaniah et al., 2019

DPPH: 2,2-Diphenyl-1-picrylhydrazyl.

### 7.4 Non-dairy beverages

Patel et al. (2023) made an herbal tea infusion using a leaf extract of *M. oleifera*. This tea was evaluated for nutrient content, and it was found to possess higher contents of iron and calcium than the commercial tea product formulations. All participating subjects were weighed and administered a sachet of the tea, which was then dissolved twice a day in 100–125 ml of hot water without sugar. Their blood haemoglobin (Hb) levels were measured in both initial (day 0) and last (day 15) samples. Compared with the initial values, Hb levels increased while body weight decreased in nearly all participants throughout the study. This means these subjects increased their Hb levels upon daily consumption of infused herbal tea of *M. oleifera* leaf extract. This can significantly elevate the concentration of Hb for the management of anaemia without inducing weight gain or loss, especially among obese subjects. According to Haque et al. (2022), drinks with 1 % *M. oleifera* extract improved health due to consistent sensory acceptability regarding colour, flavour, and taste exhibited by the leaf extract. Similarly, Jideani et al. (2021) indicated the preparation of a non-alcoholic pearl millet drink by mixing 4 % powder of *Moringa* leaf extract. The result of the study indicated volatile compounds with anti-inflammatory action. Additionally, the *supplementation of the beverage with Moringa leaf powder significantly enhanced its* nutritional value. Olusanya et al. (2020) conducted an experiment focused on the assessment of the effects of *M. oleifera* leaf powder (MOLP) on mahewu, a traditional non-alcoholic cereal beverage fermented from maize meal porridge in rural areas of southern Africa. The study aimed to ascertain the nutritional content and consumer acceptance of the beverages. MOLP supplementation significantly improved the fibre, fat, and mineral content and calcium and iron content of the beverage to a highly significant level of *p* < 0.05. The % increments of calcium and iron are found to be 350 %, 700 %, and 950 % for supplementation with 2 %, 4 %, and 6 % MOLP. The percent increment of concentration of iron is 106 %, 214 %, and 287 % for the respective percentages. Customer satisfaction decreases with an increase in the percentage of MOLP in the beverage.

Vanajakshi et al. (2015) analyzed a fermented *Moringa* leaves-based beetroot (MLBBR) drink, which was obtained from the two parts combination of beetroot juice (BRJ) with one part of the *Moringa* leaf paste. The analysis of this drink revealed a high content of live lactic acid bacteria. Apart from that, when the pH level of the beverage was set at 6.5, it stayed fresh and was safe to drink even after being chilled at 4 °C for up to 30 days. Fermentation also decreased raffinose by 60 %. Moreover, the beverage has been proven to be antimicrobial; it inhibits the activities of food-borne pathogens, including *S. aureus*, *Listeria monocytogenes*, *E. coli*, and *Bacillus cereus*. The drink had 20.79 % radical scavenging activity and a 5 mg/ml phenolic content. It also yielded 11.8 mg/ml of calcium and 0.2 mg/ml of iron. These results suggest that the fermented MLBBR beverage could well be commercially and health-benefiting.

### 7.5 Edible films/coatings

Today, a fast-growing industrial emphasis on sustainable food preservation and active packaging, which promotes the addition of bioactive compounds to understand better their effects in improving food shelf life and maintaining product integrity, is especially pertinent when aligned with the circular bioeconomy model (Barzan et al., 2024). Active packaging contains additives such as antioxidants, antimicrobial agents, ultraviolet (UV)-blockers, and other substances that can be incorporated into films. One of the most significant attributes of active food packaging is protecting food from UV rays to prevent photochemical damage. Other factors that may be considered to cause the degeneration or changes in the physiochemical and biological properties of foods come out during processing and transportation. Polymers can enhance UV blocking by using biocompatible components that absorb light within the UV range (200–400 nm) to prevent photodegradation (Ezati et al., 2023). Active packaging differs from inert packaging. Inert packaging is not reactive with food products (Jabar et al., 2023). As such, edible active packaging can become a breakthrough technology as it can be safely ingested and replace conventional packaging. Active packaging also helps maintain the sensory properties and shelf life of products (Miao et al., 2022). *M. oleifera* has been widely used in active packaging research because of the edible parts of the plant, besides its microbial and antioxidant properties (Barzan et al., 2024; Firdaus et al., 2023; Hamed et al., 2024; Nayak et al., 2024; Tesfay & Magwaza, 2017; Venkatesan et al., 2024) (Table 9).

**Table 9:** Edible films/coatings derived using different parts of *Moringa oleifera* Lam. with their applications.

Plant part used and its form	Quantity added	Matrix used	Applied on food items	Beneficial effects	References
Seed powder	1, 3, 5, and 10 wt%	Chitosan	Strawberries	The bio composite films showed significant increased antimicrobial and antifungal activity at 10.0 wt%; Effectively preserving the strawberries.	Venkatesan et al., 2024
Leaf flavonoids	0.5–4 %	Chitosan	Meat burger	Flavonoid incorporation increased antioxidation capability of the films and a 4 % flavonoids content prevented pathogenic bacteria growth in packed beef burgers	Hamed et al., 2024
Leaf extract	1 ml	Chitosan	Bread	Excellent biodegradability, anti-fogging effect, antibacterial and antifungal activity of the films were observed, minimal water solubility (over 14 days) enabling successful encapsulation in beads; Film thermal resonance increased over time and that was more efficient for storage as shelf lives of bread were prolonged	Mohamad et al., 2023
Leaf extract	0, 50, and 100 %	Pectin, and carboxymethyl cellulose	Fish	The Nile tilapia fish treated with an edible coating, especially 100 % moringa leaf extract experienced delayed decay	Firdaus et al., 2023
Leaf extract	5, 10, 20 μg/ml	Gelatin	Cheese	The wrapping of extract-loaded films with cheese underwent low variations on pH, colour and texture, and decrease in lipid peroxidation, yeasts and mold count	Mezhoudi et al., 2022
Leaf extract	10, and 20 %	Methylcellulose	Bread	The extract affected features like hardness of bread, water sorption and external growth appearance of microorganisms	Braham et al., 2022
Leaf extract	20 μg/ml	Gelatin	Fish fillet	The maximum strength of fillets was increased by 12 % after 6 days from storage; The fillets coated by gelatin-extract, had the highest sensory scores for odor, colour and overall acceptability	Mezhoudi et al., 2022
Seed powder	1, 3, 5, and 10 %	Poly(butylene adipate-*co*-terephthalate) (PBAT)	Strawberries	The PBAT-powder (1 %) containing films exhibited great performance as biodegradable packaging for strawberries, and improved storage stability against fungal infections	Verdi et al., 2021
Leaf extract	2 %	Chitosan, and carboxymethyl cellulose	Avocado	Improvement of fruit quality and shelf-life	Tesfay & Magwaza, 2017
Leaf extract	0.03, 0.05, 0.07, and 1 g	Gelatin	Gouda cheese	Better storage quality, less microbial growth, and reduced lipid oxidation was observed	Lee et al., 2016
Leaf extract	0.3 %	*Aloe vera* gel	Chicken bites	*Aloe vera* gel significantly (p < 0.05) reduced bacterial count and had better sensory scores and acceptability than untreated control.	Chauhan et al., 2016

Ranote et al., 2024 synthesized bioplastic films containing *M. oleifera* gum and polyvinyl alcohol (PVA), glycerol, and citric acid. Heating was applied to prepare this mixture, and these ingredients were cast using efficient techniques. The prepared films were tested to determine the efficacy of the synthesized film, where fresh green chillies were stored for a week. Chillies wrapped in bioplastic films retained moisture content with an unaltered original appearance, whereas the chillies without wrapping lost moisture content and became dull and wrinkled. Like the same application, Mishra and Sinha (2023) worked on *M. oleifera* seed flour as a filler in a PVA matrix to create a composite film. The synthetic biodegradable films exhibited low vapour permeability of water vapour, being 1.42 × 10^−10^ gs^−1^ m^−1^ Pa^−1^, thus suitable for various packaging applications. Bhat et al. (2022) reported that the addition of *Moringa* extract to a chitosan, guar gum, and polyvinyl alcohol (CGPM) matrix led to the creation of dense surfaces, which showed improved water resistance. In the CGPM film study, the active films presented very low water vapour permeability (WVP), oxygen permeability (OP), and total migration values, all at 10 mg/dm^2^. Moreover, these active films have been demonstrated to have inhibitory activity against *E. coli* and *S. aureus*. Chan-Matú et al. (2021) were able to conduct a study to evaluate the effect of *M. oleifera* ethanolic extract (MEE) and glycerol (G) on chitosan (C) films. Two concentrations of MEE, that is, 0.07 % and 0.15 % *w*/w and G 20 % –40 % *w*/*v*, were employed to assess the bioactive, mechanical, physical, structural, and optical properties of the films. The incorporation of MEE altered the solubility of the film.

Additionally, reduced tensile strength (TS) and elongation at break (% Eb) values by 38.3 % and 72.1 %, respectively, were determined for the samples without G and MEE. Other optical properties demonstrated that MEE is an important light-blocker in the films. Films containing MEE and glycerol had lower water vapour transmission rates (WVTR) than control samples. Among the compositions screened, those containing 0.15 wt% MEE and 40 wt% glycerol contained maximum phenolic content and antioxidant activity. According to Ju et al. (2019), infusion of Khorasan wheat starch (KWS) with *M. oleifera* leaf extract (MLE) resulted in the preparation of food packaging material possessing strong UV-blocking ability. In addition, the incorporation of 1 % MLE in KWS enhanced the biodegradability of the films observed over one month, making it a suitable choice for sustainable food packaging.

## 8 Enrichment applications of different biomasses of *M. oleifera* in feed

Due to the world's continuously increasing human population, research scientists in animal husbandry are under pressure to increase food supplies in safe, non-antibiotic-safe sources and provide an ever-increasing demand for protein and fat sources from animals with sustainable resources in feed or crop materials. Recent efforts to combat antibiotic use in livestock and poultry have become a critical initiative to address the serious problem of antibiotic resistance in developed parts of the world. Recent studies indicate that supplementing animal diets with herbs and plant extracts may support growth (Mahfuz & Piao, 2019). The application of natural medicinal plants or their extracts as feed additives in cattle production has been perceived as a potential alternative to antibiotic usage in beef production (Mahfuz & Piao, 2019). Among them, *M. oleifera* leaves are nutrient-dense and thought to be the finest supplement for feeding animals. The abundance of vitamins particularly A, B and C), rich mineral profile and high protein content, in moringa leaves make them an excellent feed option for poultry and livestock (El-Hack et al., 2018). Moreover, seed meals and leaves of *M. oleifera* serve as valuable sources of carotenoids, flavonoids, phenolics, and selenium, making them a nutritionally valuable and health-promoting ingredient with potential usage in preventive medicine (Amad & Zentek, 2023). Carotenoids are essential in animal nutrition owinf to their potent antioxidant properties (Amad & Zentek, 2023). Furthermore, *Moringa oleifera* seed extracts are rich in polyunsaturated fatty acids, significantly enhancing their nutritional profile (Ogbunugafor et al., 2011). The following subsections describe the benefits of including *M. oleifera* biomasses in animal feed formulations.

### 8.1 Chicken feed

Zhang et al. (2023) established the influence of various levels of MOLP on broiler carcass traits, growth characteristics, and faecal microbiota in two stages of growth: day 28 and day 56. Results: In the current experiment, supplementation with 3 % MOLP at the early stages of growth had a maximum positive response to growth performance with significance at (*p* < 0.05). For carcass characteristics, the highest impact on both growth stages was 5 % MOLP supplementation. MOLP supplementation at all levels resulted in significant increases relative to Bacteroides addition (p < 0.05), except in the case of the 3 % group at day 28 and the 1 % group at day 56 (Table 10). Essien et al. (2022) showed that the addition of 7.5 % of *M. oleifera* leaf meal in broiler diets did not negatively affect carcass characteristics, performance, inner organs characteristics, or lipid profiles. According to Sugiarto and Toana (2021), significant improvements in body weight gain occurred with the incorporation of 2 % to 8 % *M. oleifera* seed meal into the diet of the broilers. Body weight gain of birds in the 6 % and 8 % groups was increased the most. The 6 % and 8 % groups also showed the lowest cholesterol and fat content in the meat. Hafsa et al. (2020) demonstrated that the supplementation of broilers at concentrations of 1 % and 5 % MOL results in lower pH values in the ileum while significantly lowering the counts of *E. coli*, *Salmonella*, and *Staphylococcus* species. Moreover, MOL also supplemented the activity of antioxidant enzymes catalase (CAT), superoxide dismutase (SOD), glutathione (GSH), glutathione S-transferase (GST), and glutathione peroxidase (GPx) while substantially lowering the levels of thiobarbituric acid reactive substances (TBARS).

**Table 10:** Utilization of different parts of *Moringa oleifera* Lam. in chicken feed and their benefits.

Chicken condition	Plant part used and its form	Quantity added	Health characteristics	References
1-day-old healthy broiler chicks	Leaf powder	10 g/kg	The microbial composition of the control group and moringa-treated group was different as the treated group had 47 % increase in Bacteroides and 30 % reduction in *Firmicutes, Actinobacteria, Tenericutes and Proteobacteria*	Soundararajan et al., 2023
150-day-old healthy Cobb	Leaf powder	1, 3, 5, and 7 %	The birds fed with 3 % leaf meal displayed highest crude protein levels; When 7 % leaf meal was added to the diet, it lowered the cholesterol level in breast muscles	Mickdam et al., 2022
150-day-old healthy Cobb	Leaf powder	1, 3, 5, and 7 %	Broilers given a diet having 3 % leaf meal had higher body weight but the serum protein and albumin levels remained the same; Cholesterol, serum glucose, and triglycerides showed different levels	Alwaleed et al., 2020
10-day-old Healthy Ross-308	Leaf powder, and extract	2 and 4 g/kg (Leaf powder), 2, and 4 ml/l (Leaf extract)	Final weight, total feed intake, feed conversion ratio, increase in weight and dressing percentage were significantly (p < 0.05) improved by addition of leaf powder and extract	Hussein & Jassim, 2019
270-day-old healthy broiler chicks	Leaf powder	0.5, 1, 1.5, and 2 %	Better feed conversion and body weight gain was observed on addition of 1.5 % leaf powder	Sarker et al., 2017
100-day-old healthy Hubbard	Leaf powder	0.6, 0.9, 1.2, and 1.5 %	Addition of 1.2 % leaf powder had no effect on growth but it modified acidic mucin production and intestinal microarchitecture	Khan et al., 2017
4-Week-old healthy Potchefstroom Koekoek (PK), Ovambo (OV) and Black Australorp (BA) chickens	Leaf powder	0, 25, 50, and 100 g/kg	Black Australorp chickens were better in comparison to OV and PK strains in terms of diet usage; Leaf meals enhanced growth and carcass characteristics	Sebola et al., 2015

### 8.2 Fish feed

Emam et al. (2024) conducted research on the effect of *M. oleifera* aqueous extract (MOAE) at levels of dosages at 0, 100, 200, and 400 mg/kg feed on Nile tilapia (*Oreochromis niloticus*) for 90 days. The study measured some biochemical, haematological, and growth performance parameters. The fish that received a 200 mg MOAE/kg diet showed significant (p < 0.05) improvement in growth parameters in terms of specific growth rate and body weight, as well as enhanced haematological parameters such as packed cell volume, white blood cells, red blood cells, haemoglobin, and total serum protein from the other dosage groups (Table 11). More importantly, the 200 mg MOAE/kg feed group had a pronounced reduction (p < 0.05) in liver and renal function markers, but the lipid profile remained unaffected. Tabassum et al. (2021) established enhanced nutrient digestibility and growth in *Cirrhinus mrigala* (Mori) fingerlings fed diets based on 10 % *M. oleifera* leaf meal (MOLM). In *Clarias gariepinus* (African catfish), a 10 % inclusion of *M. oleifera* leaf meal in the diet improved the nutritional quality and growth performance of the fish well (David-Oku et al., 2018). However, a higher percentage of MOLM negatively correlated with red blood cell count, white blood cell count, haemoglobin level, and mean corpuscular haemoglobin concentration of fish (p < 0.05). Puycha et al. (2017) suggested that supplementing the diet of Bocourti's catfish (*Pangasius bocourti*) with *Moringa* leaves at a dosage of no more than 100 g/kg prevents adverse effects on growth, digestibility, nutrient utilization, and serum biochemistry.

**Table 11:** Utilization of different parts of *Moringa oleifera* Lam. in fish feed and their benefits.

Fish condition	Plant part used and its form	Quantity added	Health characteristics	References
Healthy *Clarias gariepinus* juvenile	Leaf powder	0, 10, 20, 30, and 40 %	The inclusion of 20 % leaf meal exhibited best performance of *C.gariepinus* on the 56th day	Ebuka et al., 2021
Aflatoxin-challenged Nile tilapia	Seed extract	0.5, 1 %	Seed extract supplementation at 0.5 % removed adverse effects	Abdelhiee et al., 2021
*Aeromonas hydrophila* infected Nile tilapia fry	Leaf powder	0, 15, and 50 g/kg	Nile tilapia fry with leaf powder, when administered orally led to enhanced immune responses i.e. phagocytic and lysozyme activities, respiratory burst, antioxidant enzyme activities like catalase, superoxide dismutase and glutathione peroxidase in kidneys, liver and spleen; IgM levels were also enhanced	El-Gawad et al., 2020
*Aeromonas hydrophila* infected gibel carp juvenile	Fermented leaf powder	20, 40, and 60 %	Meals prepared from fermented leaves enhance the immune response, growth, antioxidant and regulate the expression of genes related to immunity; Gibel carp developed increased resistance against *A. hydrophila* with greatest effects observed at 40 % fish meal substitution	Zhang et al., 2020
Healthy Nile tilapia juvenile	Leaf powder	0, 5, and 10 %	5 % leaf powder included in diet helped in improvement of growth, increase in body weight and improved feed conversion ratio; The serum cholesterol and triglycerides level were lower than that fed on basal diet	El-kassas et al., 2020
Healthy guppy fish	Leaf powder	0, 5, 10, and 15 %	Enhanced production of mucus in the guppy;15 % leaf powder per kg of feed is used to improve skin mucus immunity	Bish et al., 2020
Healthy gilthead seabream	Leaf powder	0, 5, 10, and 15 %	Lysozyme activities, protease, antiprotease and peroxidase which form a constituent of skin-mucosal immunity improved by 5 %	Mansour et al., 2018

### 8.3 Pig feed

Ketpanyapong and Marupanthorn (2023) demonstrated that their study was the first, providing evidence that weaned pig diets supplemented with *M. oleifera* leaf extract (MOLE) granules at 250 mg/kg should result in improved growth performance, a reduced rate of diarrhoea, and decreased microbial shedding. Chikasa et al. (2022) showed that the inclusion of 4.5 % *M. oleifera* leaf meal (MOLM) in pig diets resulted in a marked reduction in average daily feed intake compared to the 0 % MOLM diet. Moreover, Yentumi, 2021 finds no adverse effects from including 7.5 % *Moringa* leaf meal in the diets of pigs. Barman et al. (2020) reported that supplementing dried *Moringa* leaves to replace 5 % of groundnut cake in grower crossbred pigs improved growth, nutrient intake, and feed conversion ratio. Low levels, lower than 6 %, of pig diets with MOLM increased white blood cell counts and haemoglobin concentration and exhibited hypocholesterolemic effects, as reported by Serem et al. (2017).

### 8.4 Feed for grazing animals

Webb et al. (2022) assessed the consequences of supplementing *M. oleifera* leaf extract (MOLE) dry matter at 50 mg/kg concentration in South African Mutton Merino lambs for up to 23 weeks. Upon slaughter, the average weight of each lamb was found to be between 60 and 65 kg. There were no significant changes to the characteristics of the carcass concerning the introduction of extracts of plants added as feed additives in the diet of lambs. However, supplementation with MOLE led to a marked elevation of the total monounsaturated fatty acids (MUFAs) (47.3 % ± 0.66 vs 42.6 % ± 0.87, p < 0.05), oleic acid (C18:1n9c) (45.0 % ± 0.57 vs. 40.5 % ± 0.80, p < 0.05), and unsaturated fatty acids: saturated fatty acids (UFA: SFA) ratio (1.01 ± 0.03 vs. 0.85 ± 0.03, p < 0.05), which could have potential health benefits for humans. Olvera-Aguirre et al. (2020) assessed the impact of different levels of MOLE on the composition of ewe milk, milk production, and pre-weaning performance of lambs. The results of the study indicated that supplementation of hydroalcoholic extracts of *M. oleifera* leaves at dosages of 20, 40, or 60 ml/ewes/day showed no adverse effects on milk quality, milk production, or lamb performance. Moreover, substitutions of alfalfa by *M. oleifera* leaves significantly improved milk yield, milk composition, quality, and growth performance in ewes, goats, kids, and lambs (Babiker et al., 2017). Similarly, Zaher et al. (2020) reported that partial substitution of up to 25 % of dry matter concentrate feed with *M. oleifera* foliage did not adversely affect feed utilization, growth performance, mineral status, or serum metabolites profile in growing desert-raised goats kept in Abu Dhabi. Improved average daily gains were significant at or above 50 % MF levels, although at the cost of higher creatinine, blood urea, and alkaline phosphatase (ALP). Supplemental *M. oleifera* leaf at 60 g/cow/day to lactating Jersey cows also had a noticeable effect of depression in oxidative stress and improved milk production with enhanced disease resistance (Kekana et al., 2019).

## 9 *Moringa oleifera* enriched ointments

Ali et al. (2022) synthesized a wound-healing hydrogel through seed extract of *M. oleifera* in the form of hydroalcoholic extract. Hydrogels prepared with hydroalcoholic extract of *M. oleifera* seeds showed significant wound healing properties in the excision wound model that was superior to control (*p* < 0.001) and conventional therapy on the 13th day (p < 0.001). Treatment with hydrogel remarkably enhanced breaking strength in the cut model (p < 0.001). A novel hydrogel wound healing material/dressing was prepared using *M. oleifera* seed polysaccharide and polyvinyl alcohol (MSP/PVA) (Parwani et al., 2016). This material had all the key desirable properties for chronic wound treatment, including antibacterial activity, antioxidant effects, bacterial impermeability, hemocompatibility, and iron chelation. Additionally, this material was bio-degradable. It was studied in vivo using mouse models that the wound shrinkage and closure rate were higher as re-epithelialization of partial-thickness wounds occurred without scarring by 6 days. Another methanolic extract of *M. oleifera* leaves, as an ointment, treated diabetic rats infected with methicillin-resistant *Staphylococcus aureus* (MRSA) (Al-Ghanayem et al., 2022). Formulation using *M. oleifera* extract accelerated chronic wound healing by a time reduction in epithelialization, augmentation of collagen contents, capillary density, and antioxidant enzyme activities. In ethanol extraction, the leaf gel of *Moringa oleifera* was assayed for in vivo effects on palate wounds of Sprague Dawley rats (Susanto et al., 2019). Results of the study showed that maximum wound area closure was achieved with 4 % ethanolic extract. Darmaputra et al. (2022) observed that the cream prepared from a 14 % ethanol extract of *M. oleifera* leaves decreased paw edema by 27.4 % in male Wistar rats.

## 10 Safety concerns of *M. oleifera*

Experimental research for the phytotoxicity of the *Moringa* plant evaluated the phytotoxic potential of this plant and ended it with a safe profile. In one experiment, non-pregnant female Wistar albino rats were given oral doses of 2000 mg/kg of methanol-water solutions. A blood test was performed to calculate the concentration levels of total bilirubin, alanine aminotransferase (ALT), and aspartate aminotransferase (AST). The outcome of the experiment showed that the lethal dose of the aqueous extract for female rats was above 2000 mg/kg (Zhang et al., 2011). A similar study was conducted on Sprague-Dawley rats to investigate the acute toxic potential of *Moringa* leaf powder. The study also reported that the human body was nontoxic up to 2000 mg/kg from oral consumption of dried *Moringa* leaves (Adedapo et al., 2009). However, there was an alteration in studies on liver function at high dosages of methanolic leaf extracts. Awodele et al. (2012) performed a comprehensive toxicological analysis on Wistar albino mice using aqueous leaf extract of *Moringa oleifera*. In the experiment, sub-chronic toxicity was determined by oral administration of the extract at doses of 250, 500, and 1500 mg/kg per day for 60 days. Distilled water was administered to control groups. The researchers determined the LD_50_ (lethal dose, 50 %) to be 1585 mg/kg and concluded that aqueous leaf extract of *Moringa oleifera* is comparatively safe for oral administration. In contrast, Silva et al. (2024) found that administering of 1000 mg/kg of the aqueous leaf extract *Moringa oleifera* in Swiss albino mice resulted in elevation of transaminases level in both males and females. This study recommended prudence on using leaf extract of *Moringa oleifera* above 500 mg/kg for prolonged period can result in marked alterations in liver enzymes. However, the leaf extract of *Moringa oleifera* showed considerable potential as a plant preparation for regulating hyperlipidemia in mice. Acute toxicity tests declared the doses of methanol extract as safe up to 2000 mg/kg (Saleem et al., 2020). Continued treatment of the extract for five weeks at the same dosage of 400 mg/kg-bw exhibited extensive damage to the liver, thereby causing liver toxicity. Therefore, long-term intake of the methanolic *Moringa oleifera* leaf extracts is not advised as this might pose a risk of developing liver toxicity in due course (Omobowale et al., 2014). Moreover, the seed extract of *M. oleifera*, being administered as a methanolic extract, was studied for both acute and subacute toxicity in rats. Acute toxicity was noted at 4000 mg/kg, and mortality at 5000 mg/kg (Ajibade et al., 2013). In essence, the seed extract of *M. oleifera* is non-toxic for human nutritional consumption. The stem bark extract showed no adverse effects at 2000 mg/kg in acute and subacute toxicity studies. These studies have established that *M. oleifera* stem bark is safe for oral administration (Reddy et al., 2013).

## 11 Limitations of the study

The majority of the studies discussed showed successful development of various products from different parts of *Moringa oleifera* that have potential in different sectors having food, cosmetics and medicinal applications. However, there is a marked gap in literature regarding an in-depth cost analysis that would allow an accurate assessment of true market potential of *Moringa*-derived products in real world. Even though, various economic analysis platforms have provided insights into the market value of *Moringa oleifera* products but laboratory-based cost data remains unclear and underreported across the studied literature Additionally, numerous *Moringa*-based food products are commercially available. However, real market potential of such products in the context of real-world pricing, cost of production, and demand of consumer has not been effectively addressed or explored in the existing studies.

## 12 Conclusion

Underutilized crop *Moringa oleifera* biomass has proven to be a prosperous source of important micro and macronutrients and essential phytochemicals, which are highly relevant for developing bio-based metallic nanoparticles, biochar, and adsorbents. *M. oleifera* biomass also acts as a catalyst and a FAME source in biodiesel production. Phytochemicals derived from *M. oleifera* extracts enhance the flavour and overall acceptability of functional foods and animal feeds. Also, *Moringa* extracts are commercially used to make different skincare ointments. Furthermore, the administration of *Moringa* extracts in different model organisms has provided valuable insights into its safety profile.

## CRediT authorship contribution statement

**Harsh Kumar:** Writing – review & editing, Writing – original draft. **Shivani Guleria:** Writing – review & editing, Data curation. **Rajni Dhalaria:** Writing – review & editing, Data curation, Conceptualization. **Eugenie Nepovimova:** Supervision, Formal analysis, Conceptualization. **Nidhi Bhardwaj:** Writing – review & editing, Data curation. **Pooja Jha:** Writing – review & editing, Data curation. **Daljeet Singh Dhanjal:** Writing – review & editing, Data curation. **Narinder Verma:** Writing – review & editing, Formal analysis. **Tabarak Malik:** Formal analysis, Conceptualization.

## Declaration of competing interest

The authors declare that they have no known competing financial interests that could have appeared to influence the work reported in this paper.

## Data Availability

Data will be made available on request.
